# Managing an ageing cystic fibrosis population: challenges and priorities

**DOI:** 10.1183/16000617.0261-2024

**Published:** 2025-05-14

**Authors:** Freddy J. Frost, Daniel G. Peckham, Imogen C. Felton, Joanna E. Snowball, Robert D. Gray, Andrew M. Jones, Nicholas J. Simmonds, Robert W. Lord, Gregory Y.H. Lip, Hannah Chandler, Kevin Murphy, Damian G. Downey, David N. Sheppard, Jane C. Davies, Jane Bull, Paula Sommer, Belinda Cupid, Lucy Allen, Jamie Duckers

**Affiliations:** 1 Adult Cystic Fibrosis Centre Liverpool Heart and Chest Hospital NHS Foundation Trust, Liverpool, UK; 2 Liverpool Centre for Cardiovascular Sciences at University of Liverpool, Liverpool John Moores University and Liverpool Heart and Chest Hospital NHS Foundation Trust, Liverpool, UK; 3 Leeds Institute of Medical Research, University of Leeds, Leeds, UK; 4 Adult Cystic Fibrosis Centre, Royal Brompton and Harefield Hospitals, part of Guy's and St Thomas’ NHS Foundation Trust, London, UK; 5 Oxford Adult CF Centre, Oxford University Hospitals NHS Foundation Trust, Oxford, UK; 6 School of Infection and Immunity, University of Glasgow, Glasgow, UK; 7 Manchester Adult Cystic Fibrosis Centre, Manchester University NHS Foundation Trust, Manchester, UK; 8 Division of Infection, Immunity and Respiratory Medicine, University of Manchester, Manchester, UK; 9 National Heart and Lung Institute, Imperial College, London, UK; 10 Danish Center for Clinical Health Services Research, Department of Clinical Medicine, Aalborg University, Aalborg, Denmark; 11 Cardiff University Brain Research Imaging Centre, Cardiff University, Cardiff, UK; 12 Wellcome-Wolfson Institute for Experimental Medicine, Queen's University, Belfast, UK; 13 School of Physiology, Pharmacology and Neuroscience, University of Bristol, Bristol, UK; 14 Cystic Fibrosis Trust, London, UK; 15 All Wales Adult Cystic Fibrosis Centre, University Hospital Llandough, Penarth, UK

## Abstract

The increasing life expectancy of people with cystic fibrosis (pwCF), largely driven by advancements in early diagnosis, multidisciplinary care and the recent introduction of cystic fibrosis transmembrane conductance regulator (CFTR) modulator therapies, is likely to herald a shift in the focus of care toward managing the complexities of ageing. This review highlights key challenges and research priorities for addressing the health needs of an ageing CF population. A growing body of evidence underscores the heightened risks of cancers, cardiovascular diseases and changing nutritional and metabolic profiles as pwCF age. CFTR modulators have improved clinical outcomes, but their effects on inflammation, immunity and long-term disease trajectories remain incompletely understood. Nutritional management, particularly the implications of obesity and body composition, poses new challenges, as does the potential accelerated ageing of immune and pulmonary systems in CF. Emerging issues such as menopause in females with CF, lifetime antimicrobial resistance and the interplay between chronic inflammation and ageing further complicate the care landscape. The review emphasises the urgent need for multidisciplinary research programmes that integrate clinical, patient and community perspectives. Leveraging established CF registries, clinical trial networks and collaborations with ageing research frameworks is critical to addressing these challenges. Ultimately, the goal is to ensure that pwCF not only live longer but also experience improved quality of life and holistic wellbeing as they realise the full benefits of therapeutic advances.

## Shareable abstract

**Advances in the care of people living with cystic fibrosis have dramatically increased survival. Understanding how best to manage an ageing CF population is a key priority for the CF community.** https://bit.ly/4h2t3gZ

## Introduction: setting the scene for an ageing cystic fibrosis population

Cystic fibrosis (CF) is a genetic disease resulting in defective or absent CF transmembrane conductance regulator (CFTR) protein [1, 2]. Loss of CFTR function results in reduced anion flow across epithelia throughout the body [3]. As such, CF is a multisystem disease, but manifests itself most obviously in the lung, where thick viscous airway secretions and loss of antibacterial defence mechanisms trigger a cycle of inflammation, infection and progressive structural lung damage, ultimately leading to respiratory failure [4–6]. Elsewhere, the pancreas is similarly affected with endocrine and exocrine dysfunction being classic features of CF and respectively carrying significant morbidity and mortality implications. The reproductive tract, bowels, sinus and kidneys are also affected almost ubiquitously but to varying degrees of severity [7].

**FIGURE 1:**
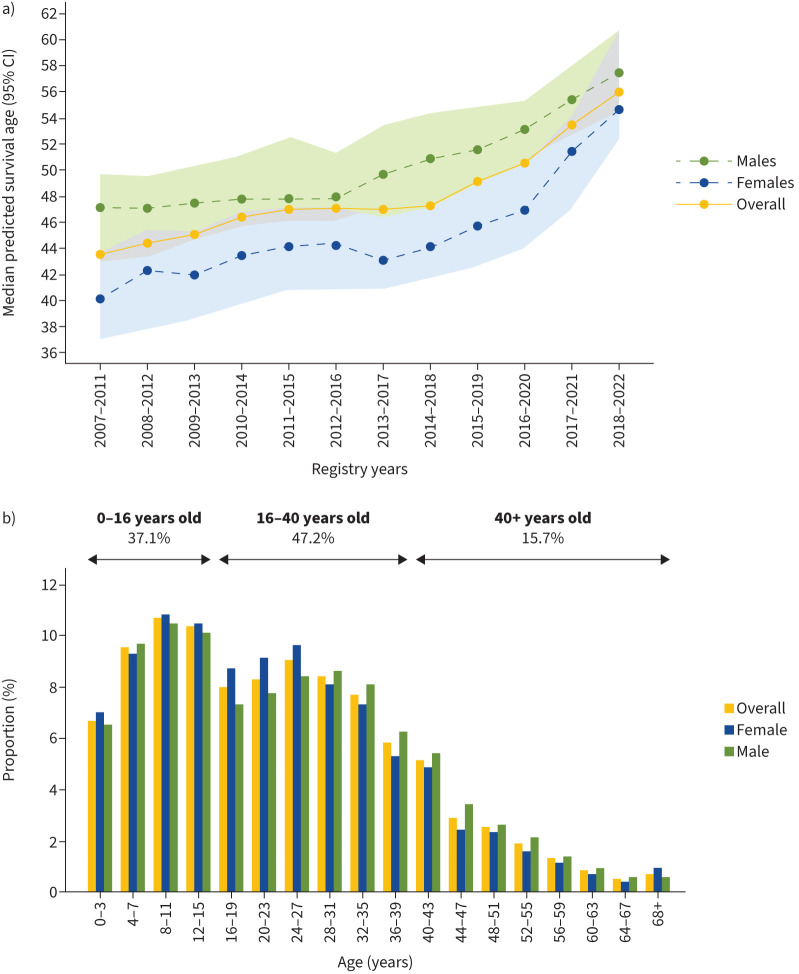
a) Temporal trends in survival for people living with cystic fibrosis (CF) and b) current age distribution for the UK CF population. Figures reproduced from [8] with permission from the Cystic Fibrosis Trust.

Life expectancy for people with CF (pwCF) has steadily increased over recent decades and in 2022 over 1600 pwCF, 16% of the UK CF population, were aged 40 years or above, see figure 1 [8, 9]. These incremental improvements in survival were achieved with a combination of early diagnoses *via* newborn screening programmes, enhanced collaborative multidisciplinary approaches allowing a focus on chest clearance and aggressive treatment of infections. Additionally, rigorous screening and optimisation of comorbidities, *e.g.* diabetes, and nutritional support have also been fundamental to improved survival [1, 10]. However, the past few years has seen a further step-change improvement in clinical outcomes for many pwCF following the licensing in Europe, North America and Australasia of elexacaftor–tezacaftor–ivacaftor (ETI) (Trikafta/Kaftrio, Vertex Pharmaceuticals) for people with the predominant *CFTR* variant F508del [11, 12]. By restoring the expression and function of F508del-CFTR, ETI has achieved significant improvements in clinical outcomes and overall wellbeing, which look set to translate into dramatically increased life expectancy for many pwCF [13]. Consistent with this idea, modelling data suggest that the median survival of pwCF (genotype: F508del/F508del) treated with ETI from 12 years is 72 years, approaching a normal life expectancy and three decades more than survival in the absence of CFTR modulators [14].

**FIGURE 2:**
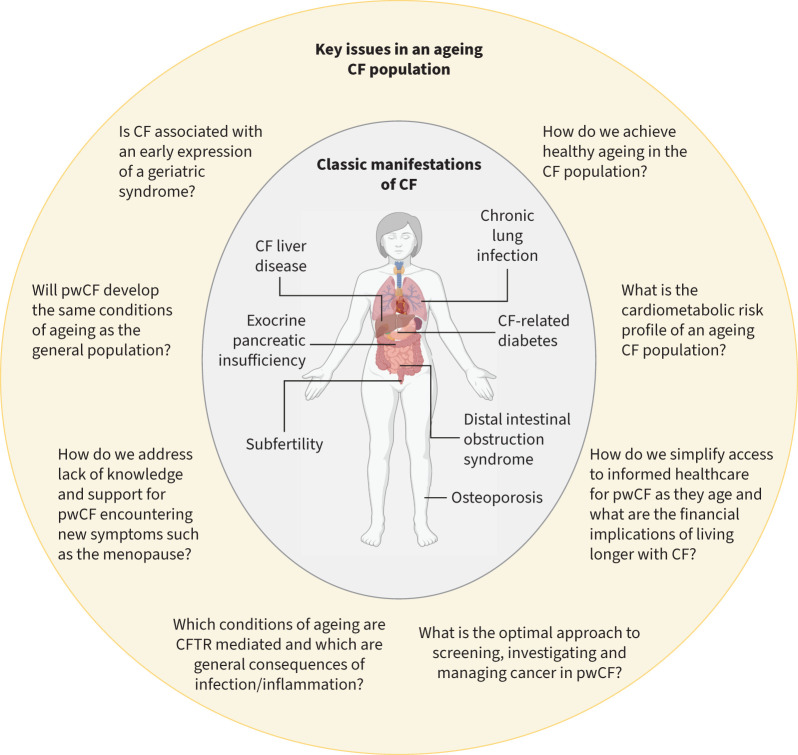
Issues and unanswered questions regarding healthy ageing in the cystic fibrosis (CF) population. CFTR: CF transmembrane conductance regulator; pwCF: people with CF.

An ageing CF population has uncertainties and challenges, see figure 2. The importance of addressing these challenges was illustrated in a recent James Lind Alliance priority setting partnership (JLA PSP), where “How do we manage an ageing population with CF?” was identified as a key research priority for the CF community [15]. A subsequent scoping review by the Cystic Fibrosis Trust identified this research priority as devoid of any established research programmes, which motivated the design and delivery of a healthy ageing with CF workshop that brought together researchers, clinicians, research funders and representatives of the CF community [16]. This article, developed following the workshop, summarises the existing evidence and knowledge gaps about the potential challenges facing an ageing CF population.

## Search strategy

In addition to the scoping review performed by the Cystic Fibrosis Trust, we searched PubMed for published research related to CF and ageing to identify relevant articles from both the pre-ETI and post-ETI literature. The subsequent included reference lists of included studies and relevant reviews were reviewed by co-authors and important omissions of highly regarded papers were added for assessment at the full-text stage.

## What new issues may become important for ageing pwCF?

### CF, ageing and cancer

Several studies report an increased risk of cancers in pwCF, especially colorectal cancer (CRC), where CF is now considered a hereditary colon cancer syndrome and CRC screening is recommended from age 40 years [17–19]. This increase in prevalence as well as the earlier onset of CRC in pwCF is likely to reflect a pro-oncogenic environment resulting from CF-related systemic and bowel inflammation, alterations in the gut microbiome and a high-fat and low-fibre diet, factors which are all associated with increased risk of CRC in the general population [20–22]. CFTR dysfunction is also highly relevant given the growing evidence that CFTR functions as a tumour suppressor gene which can alter cancer cell proliferation and metastasis [23, 24]. While the introduction of ETI may positively alter the drivers of tumorigenesis, the significant change in the demographics of the ageing CF population will result in an increasing number of individuals at risk of CRC and other age-related cancers. Furthermore, solid organ transplant recipients also have a significantly increased risk of developing a wide range of cancers when compared to the general population and this is further increased in pwCF [25].

To mitigate cancer risk as the CF population ages, there is a requirement for strategic engagement with well-established national screening programmes, including cervical, breast and CRC screening. While regional and national screening approaches will vary, CF multidisciplinary teams are well placed to use their experience in co-ordinating care across diverse specialities to ensure the integration and adoption of local screening guidelines. Specific examples of key high-yield interventions will be those targeted at improving the uptake of human papillomavirus immunisation in women with CF to minimise the risk of cervical cancer as well as skin cancer surveillance in post-solid organ transplant recipients. Research efforts are ongoing to characterise the link between CF and cancer and to identify the true risk of all types of cancers in the ageing CF population.

### Changing cardiometabolic risk profiles

pwCF have long been known to have several disease characteristics that are risk factors for cardiovascular disease (CVD) in the general population. For example, diabetes is prevalent in over 50% of adults with pancreatic insufficient CF and is associated with a more severe disease phenotype and premature death [26, 27]. Chronic inflammation, a hallmark of CF, is implicated in excess cardiovascular morbidity in diseases such as systemic lupus erythematosus, human immunodeficiency virus infection and rheumatoid arthritis [5, 28–30]. Moreover, the recommended high-fat, calorie-rich diets for pwCF, which are often supplemented with additional salt, is an additional important risk factor. Despite these risk factors, cardiovascular events for pwCF have been rare, most likely due to these risks being offset by low/normal arterial blood pressures, low body mass index (BMI) and short life expectancy [31].

With the restoration of CFTR function achieved with ETI, the balance of cardiometabolic risk may shift in the future, particularly as the CF population grows older. For example, the prevalence of hypertension increases following CFTR modulator initiation and increases in BMI continue to accelerate [32, 33]. Similarly, the prevalences of adiponectin metabolic imbalance and metabolic syndrome have also reported to be increased post-ETI [34, 35]. Conversely, CFTR modulator therapy is associated with improved glycaemic control and reduced inflammation [36–38]. However, dysglycaemia is resolved in less than half of pwCF treated with ETI, meaning potentially significantly increased lifetime exposure to the deleterious effects of diabetes for the majority [37, 39]. Similarly, inflammation is significantly reduced by CFTR modulator therapy, but inflammatory profiles are not restored to those of healthy controls, again meaning that an increased cumulative lifetime exposure to low-grade inflammation is likely [36, 38, 40].

A recent study incorporating national registry data and international electronic healthcare record data suggested that pwCF are at increased risk of CVD compared to the general population [41]. These data need prospective validation, but if correct, a key research challenge will be how best to identify those pwCF at risk of CVD to allow timely holistic risk reduction interventions. General population risk prediction tools suggest pwCF may be at excess risk of CVD; however, such tools are not always relevant to specific disease populations and new approaches may be needed [42, 43].

### Nutrition and body composition

Defining optimal nutritional status in the ageing CF population is a new challenge. The latest UK CF registry data confirm the shift in BMI category distributions. Of the 16% of adults with CF now over 40 years of age, only 7% have an underweight BMI (≤19.9 kg·m^−2^), 44% have a BMI of 20–24.9 kg·m^−2^, but 49% now have a BMI ≥25 kg·m^−2^, classifying them as overweight or obese [8].

Optimal BMI targets for pwCF of 22 kg·m^−2^ for females and 23 kg·m^−2^ for males are from registry data linking BMI to lung function [44]. However, as these data were derived two decades ago, they may not be applicable to the contemporary CF cohort [44]. Recent nutrition guidelines recommend a move away from the “sole reliance on BMI” and suggest utilising body composition measurements, which may have a stronger association than BMI with respiratory outcomes [45]. Estimating proportions of fat mass (FM) and fat-free mass (FFM) help identify pwCF with conditions such as normal weight obesity (high FM with BMI of 20–24.9 kg·m^−2^) and sarcopenic obesity (low FFM with BMI ≥30 kg·m^−2^) [46, 47]. The validity of these measurements for prognostication and the additive value of including them in registry data sets remains to be established, but is a key area of future research.

pwCF often have poor quality diets with overconsumption of fat and sugar and suboptimal fibre intake [48]. In the non-CF population, poor diet and/or obesity are risk factors for the development of age-related co-morbidities including cancers and CVD [49]. The relevance of these associations in pwCF have not yet been established, but improving diet quality is likely to be important for health outcomes independent of BMI. Changing dietary habits is challenging and potentially more so for pwCF who may have ingrained poor eating behaviours, which in the past have been positively reinforced by their clinicians [50]. A key part of the nutritional care provided to pwCF as they age will be helping them to maintain optimal body composition and weight, whilst achieving sustainable dietary change.

### Long-term consequences of inflammation

Immunosenescence, the dysfunction of adaptive and innate immune cells with ageing, is a well-described phenomenon [51]. The hallmarks of this process are thymic involution and a decline in T-cell production, leading to increased susceptibility to infection and decreased efficacy of vaccines. A further feature is inflammaging, in part due to cellular senescence and the senescence associated secretory phenotype that leads to the release of cytokines such as interleukin (IL)-6. Healthy older people over the age of 90 years have elevated cytokines, such as IL-6 and IL-1, compared to middle-aged controls, while lung ageing is associated with increased soluble and cellular inflammation [52, 53].

In CF, increased levels of pulmonary and systemic inflammation are an inherent component of the disease and higher levels of systemic inflammation are associated with worse clinical outcomes [54]. It is therefore unsurprising that cellular senescence already occurs in CF lungs beyond with the context of ageing [55]. Major questions facing the CF clinical and research community are whether the immune system in pwCF will age normally or whether this process is already accelerated and how new treatments, such as CFTR modulators, will affect immunosenescence and inflammaging as pwCF live longer. Rather than being considered in isolation, inflammaging and immunosenescence should be viewed as shared mechanisms contributing to both diseases.

What lies ahead for ageing pwCF will likely therefore much depend on the interplay between CFTR function and inflammation. CFTR influences the behaviour of inflammatory cells, such as neutrophils and macrophages, while CFTR modulators restore some function to CF inflammatory cells [56, 57]. However, it is important to acknowledge that a knowledge gap exists in how inflammation in CF will affect ageing and, conversely, how ageing will affect CF inflammation. Bold long-term observational studies will also be required to assess ageing in CF, including the measurement of inflammation not just systemically, but also in multiple tissue compartments, such as the lung and bowel.

### Infection: unknowns, lifetime exposure to antibiotics, implications for resistance

Antimicrobial resistance (AMR) is a global threat and is particularly relevant to pwCF. Indeed, a key priority in the JLA PSP was “Is there a way of reducing the negative effects of antibiotics *e.g.* resistance risk and adverse symptoms in people with CF?”.

In clinical trials, ETI achieved meaningful reductions in acute exacerbations and subsequent hospitalisations [11, 12]. Additionally, registry-based follow-up studies of earlier CFTR modulator treatments found a reduced prevalence of chronic lung infection, but approximately one third of adult pwCF remained chronically infected with *Pseudomonas aeriginosa* [58, 59]. Therefore, it is likely that a significant proportion of pwCF will have increased lifetime antibiotic exposure with important implications for off-target adverse effects, *e.g.* nephrotoxicity and ultimately AMR. The future landscape of lung infection in CF is therefore unclear and maintaining robust surveillance for new infections will be challenging. Thus, new approaches to diagnosing, managing and mitigating AMR are likely to be needed with the potential for fresh perspectives on historical practices such as dual and prolonged antipseudomonal antibiotics for acute exacerbations. Ongoing efforts such as the STOP-360 clinical trial programme in North America and the LifeArc/CF Trust Hub Network in the UK are seeking to address this challenge and redefine optimal antimicrobial strategies in the post-modulator era.

### Menopause

An ageing CF population will manifest an increased proportion of females with CF (fwCF) experiencing menopause. This phase of life has profound impacts on both short- and long-term health and wellbeing, but there is a critical lack of evidence about its interplay in CF and is currently poorly understood and under-investigated [60]. Whilst the average age of menopause (defined as 12 months from final menstrual period) is 51 years in the general population, it has been reported to be earlier, at 48 years in CF [61, 62]. The approximately 10-year phase leading up to the menopause is termed perimenopause [63]. By implication, this menopausal transition phase may begin for fwCF in their late 30s, with as yet unknown effects on prospective CF-aging experiences or outcomes. A key area of concern is bone mineral density (BMD), given that osteoporosis and osteopenia are classical manifestations of CF and accelerated BMD loss and increased risk of fracture are seen during the menopause [64–66].

FwCF recently cited effects of sex hormones on CF and menopause as within their top five priorities for sexual and reproductive health support and research [67]. This is likely driven by the burden of symptoms experienced by fwCF, where symptomatic manifestations include early wake-up (83%), stiffness or soreness in joints, neck or shoulders (65%), night sweats (65%), hot flashes/flushes (58%) and vaginal dryness (54%) [61]. Importantly, there is a significant crossover of symptomatology with the features of CF-associated nontuberculous lung infections, CF-arthropathy and CF-pelvic floor morbidity, such as stress-urinary incontinence. Additionally, from this same study, nearly one-third of fwCF experienced worsening of CF symptoms during perimenopause, whilst 42% of fwCF reported experiencing worsening CF symptoms on reaching menopause [61].

Established longer-term benefits for women in the general population starting hormone replacement therapy within 10 years of their menopause include reduced risk of osteoporosis and osteoarthritis, CVD, type 2 diabetes, depression and dementia [68–72]. Understanding the CF-specific experience and potential of beneficial risk-reduction for fwCF who have an *a priori* excess risk of ageing complications with close overlap to those described above may therefore highlight modifiable therapeutic targets.

Ultimately, the overall ambition must be to empower fwCF to experience optimal health and wellbeing as they age with CF. Research is needed to define the optimal scope of care-model provision, while practical implementation will likely require the development of inter-specialty care guidelines in collaboration with fwCF, CF clinicians, menopause specialists and primary care providers.

## What does ageing with CF mean: a community perspective?

The JLA PSP refresh of the research priorities for the CF community in 2022 revealed that ageing in CF is a research priority for the more than 1300 global CF community members who took part [15]. This was strengthened by a Cystic Fibrosis Trust community involvement team facilitated focus group with a diverse group of older pwCF.

“Living longer is a wonderful gift, but also needs to be lived well.”

The group highlighted that the increasing diversity in age in pwCF brings diversity in health history and comorbidities; no two pwCF at age 50 years will have experienced the same trajectory. With the prospect that there could be more years than expected ahead of them, comes the need to preserve the longevity they never anticipated experiencing. One pwCF, told us “Living longer with a chronic condition is exhausting needing appropriate guidance and support”.

Thus, the ageing CF population experiences a great deal of uncertainty. Key areas identified include greater understanding of what ageing changes can be attributed to either the impact of CF or the impact of the medications and procedures that pwCF have endured so far.

“There's no room for complacency.”

For example, pwCF might be unable to decipher, whether the joint pain or inflammation that they experience are appropriate for their chronological age or exacerbated at an earlier age due to CF or CF medication. A fwCF highlighted some of these complexities by expressing “Is feeling hot in the night due to the start of a CF lung infection, a CF diabetes hypoglycaemia or is it a hot flush due to the menopause?”.

“My CF team thought hormone replacement therapy would be OK, but my GP refused”.

Concerns emerged regarding the understanding of CF by other specialties, healthcare services and social care infrastructure. Health passports for pwCF alongside an education of aligned specialties were highlighted as potential strategies to address these concerns. More broadly, there was support for the need to capitalise on existing health data and ensure that sample and data collection are maximised for current and future research to minimise the burden on pwCF.

## Strengths in CF research that could be applied to ageing research?

The CF research community has strengths which might be leveraged for high-quality research programmes about ageing in CF. These include expertise in a wide range of fundamental scientific disciplines and abundant well-validated *in vitro* and *in vivo* models of CF disease that could be repurposed towards pertinent questions relating to the pathophysiology of ageing in CF. The understanding of systemic and airway inflammation in pwCF receiving CFTR modulator treatment and their long-term sequelae is an area that the CF research community is particularly well equipped to answer.

Well-established population-level registries are an invaluable resource for detailed epidemiological studies of CF [73]. CF registries are usually organised at a national level and consist of either encounter or annual based data entry. Careful consideration as to the data required to answer the key epidemiological questions in an ageing CF population is needed to ensure optimal standardisation and efficiency of data collection without unduly burdening CF clinical teams. An international collaboration led by the Cystic Fibrosis Registry Global Harmonization Group delivered important insights into the outcomes of COVID-19 for pwCF during the pandemic and could serve as a blueprint to enhance standardisation and accelerate research in the setting of ageing [74].

In addition to registry collaborations, established clinical trial networks exist at the national and international level. These networks were vital to the efficient recruitment and delivery of modulator studies and provide excellent infrastructure for future rapid delivery of industry and investigator-led programmes.

As highlighted in the Introduction, much of the improvements in CF clinical care prior to the development of CFTR modulators were delivered through multidisciplinary team approaches which already have experience in managing multimorbidity and polypharmacy and are therefore already well positioned to manage an ageing population. Refining these multidisciplinary teams to include expertise in the management of potential emergent ageing syndromes will allow rapid translation of research-derived insights towards clinical practice.

### Current areas of weakness/opportunity/growth?

In the past decade, there has been a dramatic increase in the wider promotion and delivery of ageing research with national (*e.g.* UK Ageing Network, www.ukanet.org.uk) and international ageing networks (*e.g.* EuroAgeNet, www.liverpool.ac.uk/life-course-and-medical-sciences/research/euro-age-net/) working collaboratively to build capacity and stimulate advances that address health resilience and quality of life in older age. However, until recently, there has been little focus on the interplay between ageing and CF. Illustratively, a scoping review by the Cystic Fibrosis Trust identified no major ongoing research grants in this area [16].

There is a spectrum of areas that need urgent attention. A key question is the rate of ageing in the CF population. Accelerated ageing syndromes are well-recognised and rapid ageing has been associated with lower lung function in non-CF populations [75]. A key priority is to understand the phenotypic age of pwCF and how it is influenced by CFTR modulator therapy. Other more focused questions include the risk of neurodegenerative diseases of ageing for pwCF, who may carry a number of risk factors for these diseases, such as chronic inflammation, diabetes and hearing loss [76]. Indeed, abnormal brain imaging has been observed in CF, but its consequences for long-term outcomes in an ageing population are unclear [77, 78]. To address these questions about ageing with CF, the CF research community must now embrace opportunities to collaborate with ageing networks. In particular, the development of CF models suitable for ageing research is a key objective. For two reasons, *Drosophila melanogaster* might be a suitable model. First, *Drosophila*’s short lifespan, genetic tractability and amenability to powerful imaging techniques make it a widely used model organism for ageing research [79]. Second, a CFTR orthologue has been identified in *Drosophila*, knockdown of which in the adult *Drosophila* intestine led to a CF-like phenotype, which was rescued by expression of human CFTR [80]. With this and other CF animal models, new insights into ageing in pwCF will likely be achieved [81, 82].

## Summary

Transformational changes in the prognostic outlook of pwCF have been achieved in recent years and CFTR modulator treatments are likely to further increase life expectancy in the next decade. However, an ageing CF population with shifting clinical needs leaves many unanswered questions and at present there is little established research in this area. Collaborative, multi-disciplinary research programmes incorporating academia, clinical teams and the CF community itself are needed to ensure pwCF realise the full benefits of their newly achieved prolonged lifespan.

**Points for clinical practice**

- Advances in the care of people living with CF have dramatically increased survival.
- People living with CF may be high risk for some cancers and cardiometabolic diseases as they grow older.
- Understanding how best to manage an ageing CF population is a key research priority for the CF community.
